# A recipe for mediocrity and disaster, in five axioms

**DOI:** 10.7554/eLife.01138

**Published:** 2013-07-16

**Authors:** Henry R Bourne

**Affiliations:** Department of Cellular and Molecular Pharmacology, University of California at San Francisco, San Francisco, United States. He blogs about the challenges and opportunities associated with biomedical research at biomedwatch.wordpress.com. His research was supported by the NIH from 1969 to 2008, and he served as a reviewer of grant applications at intervals during this period. henry.bourne@ucsf.edu

**Keywords:** point of view, science policy, careers in science, NIH, postdoc, grad school, funding

## Abstract

Biomedical research in the US will become unsustainable unless scientists and research institutions start to question certain assumptions they have long taken for granted.

Scientists revel in new discoveries, especially when they overturn received wisdom. Paradoxically, however, scientists show little interest in changing how they go about their work. Consequently, the iron grip of habit and outdated assumptions controls their labs, institutions, socio-economic behaviours and future prospects. Moreover, as I have argued before, unless scientists and the institutions that employ them make radical changes in the near future, the biomedical research enterprise in the United States will become unsustainable (Bourne, 2013). Scientists in the rest of the world would be advised to pay attention to what is happening in the US and to avoid repeating these mistakes elsewhere.

Five axioms underlie the unsustainable state of US biomedical research, and are accepted as self-evident truths by many scientists and institutions. However, as I explain below, persistent adherence to these axioms is leading the whole enterprise to certain disaster.

## Axiom 1: bigger is always better

This axiom, an article of faith in many parts of the modern world, is at least partly responsible for the economic depression that started in 2008 and is still very much with us. The unsustainable nature of biomedical research is, comparatively speaking, small potatoes, but its roots are similar. The predicament of researchers today grew out of decades of dreaming about, and eventually achieving, bigger labs, bigger grants, and bigger research universities, medical schools and biomedical research institutes (Bourne, 2013).

Why is big always better? A common answer: ‘If we don’t expand, we die’. To some degree, this may be correct for the business of delivering medical care, which depends on economies of scale and large patient populations that include the young and healthy. But does the expand-or-die dictum apply to all biomedical research? Up to a point, certainly: large institutions allow greater numbers of principal investigators (PIs) to collaborate, to share knowledge and costly equipment, and to attract first-rate trainees and prospective PIs. Nonetheless, large institutions also suffer from real disadvantages. Visiting one highly respected mega-institution, I met two scientists who did not know that their labs were doing the same experiment, although they worked in the same building—and did not even know each other’s names. In addition to hampering communication, bigness can endanger an institution’s vitality by damaging community morale, support for young faculty, and maintenance of uniformly high research standards. I suspect that bigness also poses similar problems at the level of individual research groups and laboratories, and that productivity per unit of funding or per working scientist starts to fall when the group or laboratory increases above a certain size (Hubel, 2009; Berg, 2012; Fortin and Currie, 2013).

Bigness can endanger an institution’s vitality by damaging community morale, support for young faculty, and maintenance of uniformly high research standards.

In either case, part of the problem stems from an over-reliance on quantity, which can be measured easily (in, for example, terms of grant income, lab square feet or numbers of research faculty), at the expense of quality, which is harder to assess. We really need to know which ways of organizing and funding research best enable scientists to pose important questions and produce valuable new understanding.

So, what can researchers do in their own institutions to make the research enterprise more sustainable, and to ensure that quality is valued at least as highly as quantity? The answer is to do what you normally do in the lab—that is, follow your curiosity. Start with those parts of your department or institution that you know best and explore which efforts deserve, on grounds of quality, to be expanded, which should be maintained at their current levels, and which should be reduced (or scrapped altogether). Test your conclusions by asking more questions, gathering further information and making comparisons with other parts of the department or institution (see Box 1 for more details). And when you have convinced yourself that you know how to improve the overall quality of research, find allies and make it happen.

Box 1. **Questions to explore at your own institution.**

Scientists often think that they know how their department or institution works, where its defects are, and what needs to be done to fix them. On further examination, however, they may discover that they know less than they think. Thus the first step towards reform is to ask questions and get reliable (and, where possible, quantitative) answers. Answers to the questions below will help you learn a lot more about your department or institution.

**Money.** Where does the money come from (e.g., federal grants, indirect cost reimbursements, soft-money salaries, support for graduate students)? Where does it go, and who decides where it goes?

**Organization of research labs.** How many PIs direct research labs? How big are these labs in terms of people and funding? How many graduate students, postdocs, technicians, and staff scientists are there per lab? How are these and other resources (e.g., equipment) distributed? Who makes those decisions, and on what criteria?

**Research training.** How are graduate students and postdocs chosen? What skills do graduate students and postdocs learn? And what careers do they pursue after they leave the institution?

**Research faculty.** How many work in your department or institution? How are they distributed with respect to age, salary, academic rank, research support, teaching responsibilities, and recognition (prizes, honours. etc)? How much of their salary is paid by the institution, and how much comes from grants and other sources?

**Quality vs. quantity.** Begin with faculty scientists you know well: how do your subjective estimates of their scientific ability compare to quantitative measures (such as publications and income from grants)? Using the latter measures, compare faculty in different departments. How does your institution judge quality?

## Axiom 2: top-down always wins

For the biomedical research enterprise, this axiom is a lethal enemy of necessary change. ‘Yes, you are right, but X won’t let that happen’. At the National Institutes of Health (NIH) in the US, X can be Congress, the NIH director, or another agency official. In research institutions, X can be a provost or dean, a philanthropic donor, human resources, or stick-in-the-mud colleagues. And in the lab, X can be the animal care facility, the biological safety office, the NIH, journal editors, or the other scientists who review our papers and grant applications (and sometimes, sadly, the PI).

Top-down does not automatically have to win, but it usually does because bottom-up petitioners don’t speak with sufficient clarity, force, stubbornness and volume. For example, the NIH pays lots of attention to what institutions and PIs want. Moreover, it is not true that bottom-up arguments are always doomed to fail within modern biomedical research centres. Yes, officials at such institutions can be dazzled by indirect cost payments and expansive research initiatives, while also being ignorant of what is needed to perform high-quality science. But in the real world many administrators *do* sincerely support first-rate science: the problem is that they are forced also to spend most of their time putting out fires that have little to do with science.

Moreover, when institutions do not support excellent science, scientists are at least partly at fault for failing to communicate a clear vision of biomedical research and what it contributes to—and needs from—a university or medical school. These failures sometimes reflect our wilful inattention to the problems of unsustainability, which we as scientists helped to create, plus generous dashes of arrogance, entitlement and ignorance of institutional missions that do not directly benefit us.

It will not be easy for PIs to persuade their institutions to change, or for institutions to persuade funding agencies to change, but if PIs and institutions remain silent, nothing good is likely to happen, and continuing with the status quo is not a happy prospect.

## Axiom 3: the philanthropist is always right

As the biomedical research community in the US discovered it could not rely on ever-increasing levels of funding from state and federal government, institutions sought other sources of support. Industry funds some kinds of academic biomedical research, but limits its investments to protect its profits. As billionaires grow richer in a still-stagnant economy, the obvious alternative source is philanthropy. This is not news for many academic institutions, but recently, as government support became less and less generous, state universities and medical schools in the US joined their private counterparts in a vigorous pursuit of philanthropy (Mervis, 2013).

Is the philanthropist always right? I ask because in recent years many institutions have built large biomedical research facilities, often named after philanthropists (Bourne, 2013). But many of those facilities have proved to be risky investments because it has been difficult to find enough researchers, who are reliably successful when applying for grants, to fill them. I have been told that most present-day philanthropists prefer to spend their money on buildings that are named after them, rather than on named professorships, but a senior fundraiser at Johns Hopkins University recently expressed the opposite opinion in an interview with *Science*: ‘most donors don’t want to see their names on a building. They think the institution should be responsible for the physical plant. They want to support people’ (Mervis, 2013). Here intuition suggests that institutional leaders themselves may often have opted for construction money, rather than support for scientists and their research, because they felt sure that NIH dollars would continue to increase. However, the days of significant increases in public funding for biomedical research are over, in the US at least. Now universities need money to pay their professors more than they need money for new buildings. It is vital, therefore, that researchers make this crystal clear to leaders of the institutions where they work, and that this message also reaches prospective philanthropists.

Researchers and fundraisers also need to persuade more philanthropists to fund research into basic biological mechanisms rather focus almost exclusively on specific diseases. Although I have not met many philanthropists, one example at the University of California, San Francisco (UCSF)—where I have spent most of my career—is instructive. Herbert and Marion Sandler approached UCSF, more than 15 years ago, seeking to invest in areas of research where additional money might be leveraged into transformative discovery. They funded research that was too risky for the NIH to fund, with a remarkable result: a seven-fold return on their investment, in the form of subsequent federal grants. We need philanthropists who think like the Sandlers. Send out the search parties, now!

## Axiom 4: only active researchers can teach science to medical students

Fifty years ago, my ‘basic science’ teachers in medical school directed active laboratories. Since then I have been told many times that active scientists are the only people adequately qualified to teach basic biomedical science—biochemistry, physiology, anatomy, microbiology and pathology—to medical students. For several decades I relayed this message to others, but over the past 20 years I have learned, along with many of my colleagues, that this is balderdash. Yes, some excellent investigators are superb teachers, but research luminaries can also prove pedestrian and sometimes execrable teachers of medical students. Indeed, many of the very best ‘basic science’ teachers at UCSF—PhD holders who have a broad, deep knowledge of biomedical science—have not worked in a lab for decades. Like the other axioms cited above, this axiom is wrong. And so, I think, is the related axiom that a medical school can only produce superb doctors if it has a stellar research faculty.

It is important to abandon these two axioms now for the following reason: the US needs more doctors, so a number of new medical schools are being planned and built; to attract new jobs and prosperity (in the form of biotech companies) to the surrounding area, these new medical schools will build labs and hire researchers; however, this will also lead to more applicants for research grants from a funding pot that has stopped growing. In other words, adhering to these mistaken axioms about teaching and research will lead to another triumph for quantity over quality. In short, it will be a sure-fire recipe for mediocrity.

Many researchers, physicians and leaders in US medical schools know in their hearts that these axioms about teaching and research are wrong, so why don’t they say so in public? Partly, I suspect, they are still in thrall to the bigger-is-better axiom: a big, highly successful research programme enhances a medical school’s prestige and helps to attract students, faculty, patients and philanthropic donors, so schools with big, prestigious research establishments consider it impolitic to admit that research labs don’t enhance medical education very effectively. Why, though, do budding medical schools and those with weak research programmes stick to the mantra? I suspect the glitter of large research centres simply misleads them, and they hope that the writing on the wall is not true.

Certainly we need to create new medical schools to train more doctors, and we should continue to teach medical students the key elements of biomedical science. Moreover, professors in a medical school, even those who are primarily researchers, should teach medical students. But that doesn’t mean every medical school must maintain a major research programme. Most important, we need to recognize that students often learn more from superb teachers than from scientists who are focused on their own research.

We need to recognize that students often learn more from superb teachers than from scientists who are focused on their own research.

## Axiom 5: trainees equal laboratory workforce

This axiom was ideally suited to the early years of biomedical research, when expanding the enterprise by making new researchers was almost as valuable for the public good as making new discoveries. But today the widespread practice of equating trainees with laboratory workforce contributes greatly to the unsustainability of biomedical research, making axiom 5 more dangerous than useful. I will discuss this in more detail in a later essay.

## Perspective

All five of these axioms pose serious dangers for the biomedical enterprise, for the same reason. From the 1950s until the end of the 1970s, the axioms worked well because national conditions were ideally suited to rapid expansion. The NIH budget kept growing and new discoveries came fast and furious, opening up new areas of science and bringing new treatments and ways to prevent disease. Bigger was better, top-down was useful for getting things done, philanthropists were (nearly) always right, and it made good sense for growing numbers of biomedical scientists to teach medical students. Now, none of the axioms really fits prevailing conditions or the quite different challenges we face. To meet those new challenges, we must free ourselves from the old axioms and devise a radically different model for biomedical research.
